# New Inducible Nitric Oxide Synthase and Cyclooxygenase-2 Inhibitors, Nalidixic Acid Linked to Isatin Schiff Bases via Certain l-Amino Acid Bridges

**DOI:** 10.3390/molecules21040498

**Published:** 2016-04-15

**Authors:** Ahmed M. Naglah, Atallah F. Ahmed, Zhi-Hong Wen, Mohamed A. Al-Omar, Abd El-Galil E. Amr, Atef Kalmouch

**Affiliations:** 1 Department of Pharmaceutical Chemistry, Drug Exploration & Development Chair, College of Pharmacy, King Saud University, Riyadh 11451, Saudi Arabia; malomar1@ksu.edu.sa (M.A.A.-O.); aeamr1963@yahoo.com (A.E.-G.E.A.); 2 Peptide Chemistry Department, National Research Centre, 12622-Dokki, Cairo 12311, Egypt; kalmoush@yahoo.com; 3 Department of Pharmacognosy, College of Pharmacy, King Saud University, Riyadh 11451, Saudi Arabia; 4 Department of Pharmacognosy, Faculty of Pharmacy, Mansoura University, Mansoura 35516, Egypt; 5 Department of Marine Biotechnology and Resources, National Sun Yat-sen University, Kaohsiung 804, Taiwan; wzh@mail.nsysu.edu.tw; 6 Applied Organic Chemistry Department, National Research Centre, 12622-Dokki, Cairo 12311, Egypt

**Keywords:** nalidixic acid, isatin, l-amino acid, Schiff base, iNOS, COX-2, anti-inflammatory, microwave irradiation

## Abstract

A series of new Schiff bases were synthesized by condensation of isatins with the nalidixic acid-l-amino acid hydrazides. Prior to hydrazide formation, a peptide linkage has been prepared via coupling of nalidixic acid with appropriate l-amino acid methyl esters to yield **3a**–**c**. The chemical structures of the new Schiff bases (**5b** and **5d**–**h**) were confirmed by means of IR, NMR, mass spectroscopic, and elemental analyses. The anti-inflammatory activity of these Schiff bases was evaluated via measurement of the expressed inducible nitric oxide synthase (iNOS) and cyclooxygenase-2 (COX-2) in the lipopolysaccharide (LPS)-stimulated RAW264.7 macrophage cells model. The Schiff bases exhibited significant dual inhibitory effect against the induction of the pro-inflammatory iNOS and COX-2 proteins with variable potencies. However, they strongly down-regulated the iNOS expression to the level of 16.5% ± 7.4%–42.2% ± 19.6% compared to the effect on COX-2 expression (<56.4% ± 3.1% inhibition) at the same concentration (10 μM). The higher iNOS inhibition activity of the tested Schiff bases, relative to that of COX-2, seems to be a reflection of the combined suppressive effects exerted by their nalidixic acid, isatins (**4a**–**c**), and l-amino acid moieties against iNOS expression. These synthesized nalidixic acid-l-amino acid-isatin conjugates can be regarded as a novel class of anti-inflammatory antibacterial agents.

## 1. Introduction

Since the introduction of the first quinolone, nalidixic acid (1-ethyl-1,4-dihydro-7-methyl-4-oxo-1,8-naphthyridine-3-carboxylic acid) for clinical use in 1967, structural modifications have continued and resulted in five generations of quinolones with an improved spectrum of antimicrobial coverage and pharmacokinetics [1,2]. Moreover, chemical modifications of nalidixic acid are still working to develop new macromolecules with enhanced therapeutic properties to combat the emergence of multidrug-resistant bacteria [3]. Microbial infections cause inflammation that attract and activate phagocytes (e.g., neutrophils) and induce immunity against the infectious agents [4]. However, the consequent release of inflammatory mediators together with the increased production of oxygen species contributes to the killing of bacteria but also damages the surrounding tissue. Therefore, resolution of the acute inflammatory response is crucial to avoid excessive damage to tissues [5]. Sulphasalazine represents the best example of an antibiotic that became an anti-inflammatory drug. Mounting evidence has shown inhibitory effects of antibiotics, belonging to macrolides, tetracyclines and quinolones, on the generation of inflammatory mediators and on immune responses [6,7]. The antibacterial 4-quinolone derivatives such as ciprofloxacin, tosufloxacin, levofloxacin and moxifloxacin, and other quinolone substituted thiazolidin-4-ones, were recently proposed as anti-inflammatory agents [8,9], and some could significantly protect mice injected with a lethal dose of LPS against death. It is also known that isatin has exhibited wide antimicrobial activity [10,11] and has been reported, along with its 5-, 6-, and 7-substituted analogs, to inhibit the production of inflammatory mediators [12]. Moreover, certain branched amino acids (BAAs) such as Isoleucine, Leucine, and Valine have been found to exhibit *in vitro* and *in vivo* anti-inflammatory activity [13,14]. An improvement for the anti-inflammatory effect of some compounds, including nonsteroidal antiinflammatory drugs (NSAIDs), was also manifested when N-linked with certain amino acids [15,16,17]. Besides that, a selective COX-2 inhibitory activity [18] was a beneficial effect. 

In this work, a series of nalidixic acid-based conjugates were constructed from l-amino acid and an isatin analog through carboxamide, hydrazide, and Schiff base formation, successively, with the aim of modification and potentiation of the biological activity of naldixic acid nucleus. The carboxamide was done using one of three naturally occurring amino acids (l-Val, l-Leu, or l-Phe) through the amidation of the carboxylic group at the 3-position of the nalidixic acid molecule by mixed anhydride coupling methods [19]. Our preliminary antibacterial screening of these nalidixic acid-based conjugates has shown a wide spectrum antimicrobial activity relative to nalidixic acid itself [20]. However, on the basis of the previously reported anti-inflammatory activity of certain amino acids [13,21] and isatin analogs [12], the anti-inflammatory activity of the new nalidixic acid-based conjugates containing these moieties were thus evaluated *in vitro*. The anti-inflammatory activity of the conjugates was determined in comparison to their individual moieties via measurement of the expressed pro-inflammatory iNOS and COX-2 proteins, using the LPS-stimulated RAW264.7 macrophage cells model [22].

## 2. Results

### 2.1. Chemistry

**Scheme 1:**
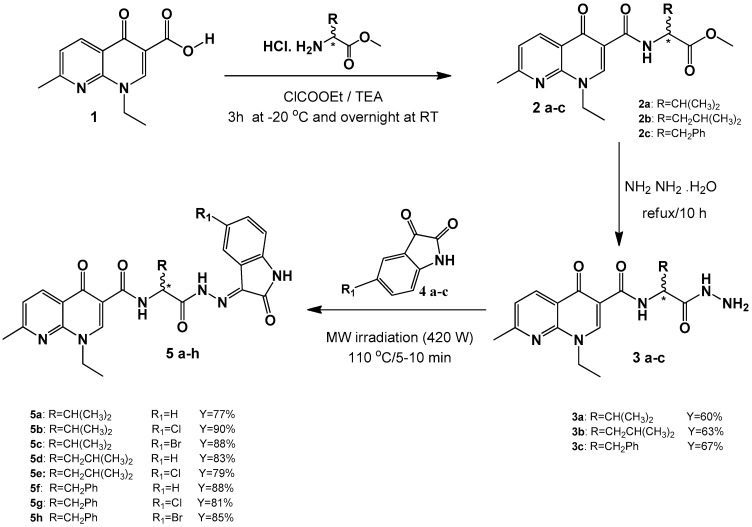
Synthetic routes for compounds **3a**–**c** and **5a**–**h**; Y = yield.

The strategy for this work is to construct a linear nalidixic acid-based conjugate that possesses a carboxamide with amino acid and a Schiff base of indoline-2,3-dione (isatin) moieties that may add new biological activity for nalidixic acid. The selection of isatin and appropriate l-amino acid moieties has been considered on the basis of their previously reported anti-inflammatory effects and the antimicrobial activity of istains. By the mixed anhydride method [23], the methyl esters of l-Valine (Val), l-Leucine (Leu) and l-Phenyl alanine (Phe) have been coupled smoothly and quickly, by mixed anhydride method, with nalidixic acid to yield the corresponding nalidixic acid amides (**2a**–**c**) in high yield. Hydrazinolysis of **2a**–**c** with hydrazine hydrate afforded the corresponding hydrazides (**3a**–**c**), which were then condensed with isatin analogs (**4a**–**c**), in acidified ethanol under microwave irradiation (420 W/110 °C/5–10 min), to give the corresponding Schiff bases (**5a**–**h**) in a high yield and high purity (Scheme 1). Microwave-assisted Schiff bases synthesis has been accomplished in a small amount of solvent and very short reaction time (5–10 min) with a high yield of pure products. Microwave irradiation had been reported to trigger heating to carry out the chemical reaction by dipolar polarization and ionic conduction [24]. Finally, the chemical structures of the hydrazides (**3a**–**c**) and final conjugates (**5a**–**h**) were established on the basis of their mass spectroscopy (MS) and NMR spectral data.

### 2.2. The Anti-Inflammatory Activity

LPS is a major component of Gram-negative bacteria cell walls, which have been implicated in septic shock. This is accompanied by an overproduction of inflammatory mediators such as NO and prostaglandins [25,26], which resulted from the expression of iNOS and COX-2 proteins, respectively. Therefore, compounds capable of reducing/blocking LPS-induced expression of iNOS and/or COX-2 proteins might be beneficial in management of the inflammatory responses. In this study, an *in vitro* assay of LPS-treated RAW264.7 macrophage cells linked with immunoblot analysis was carried out to identify the iNOS and/or COX-2 inhibitory compound. Dexa–methasone (10 μM), a well-known anti-inflammatory drug and an inhibitor of iNOS and COX-2, [27,28,29] was used as a positive control. Prior to carrying out the anti-inflammatory assay, the cytotoxicity of isatins (**4a**–**c**), nalidixic acid (**1**) and conjugates (**3b**, **3c**, **5b**, **5d**–**h**) was measured at the same concentration (10 μM) against RAW264.7 macrophage cells, via Alamar Blue assay. The compounds showed no cytotoxicity (cell viability >90%).

#### 2.2.1. The Effect of Amino Acids and Isatins on iNOS and COX-2 Expression

**Figure 1:**
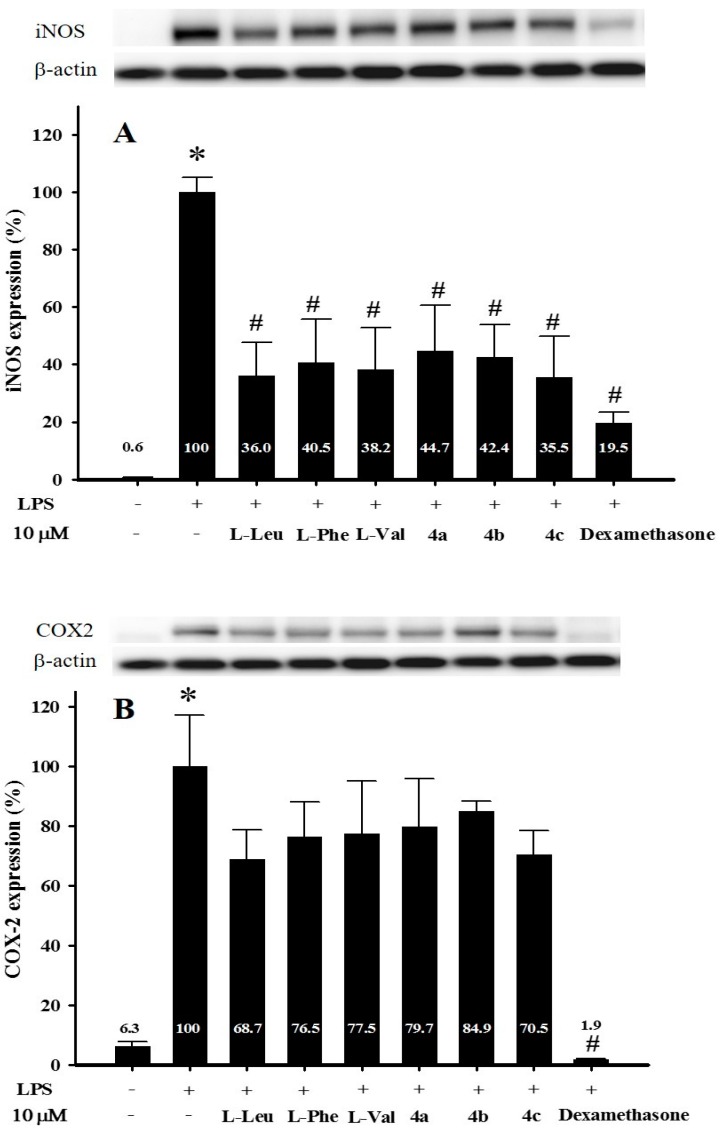
Effect of the amino acids l-Leu, l-Phe, and l-Val; and isatin derivatives **4a**–**c** and on (**A**) iNOS and (**B**) COX-2 proteins expression of LPS-stimulated RAW264.7 macrophage cells by immunoblot analysis. The results are expressed in means ± SEM * *p* < 0.05 and # *p* < 0.05, as compared to the control and LPS-stimulated group, respectively. β-Actin was used as protein equal loading control.

The amino acids (l-Val, l-Leu, and l-Phe), isatin (**4a**) and halogenated istains (**4b** and **4c**), which are incorporated in the newly synthesized nalidixic acid-based conjugates (**5a**–**h**), had then been screened for iNOS and COX-2 inhibitory activity. It has been found that the tested l-amino acids strongly, selectively, and significantly (*p* < 0.05) reduced the level of iNOS protein (Figure 1) to the range of 36.0% ± 11.5%–40.5% ± 15.1% at 10 μM, relative to that (100%) of the control LPS-stimulated cells, which was nearly half the potency of dexamethasone (19.5% ± 3.86% inhibition). However, their effect on LPS-induced COX-2 expression was insignificant. In the same experiment, isatin (**4a**) and its halogenated analogs: 5-chloro- and 5-bromo-2,3-dihydro-1H-indole-2,3-dione (**4b** and **4c**, respectively) also selectively down-regulated the iNOS protein expression to 44.7% ± 15.9%, 42.4% ± 11.5%, and 35.5% ± 14.2% at 10 μM, respectively, in increasing order of potency (Figure 1).

#### 2.2.2. The Effect of Nalidixic Acid and Its Conjugates on iNOS and COX-2 Expression

**Figure 2:**
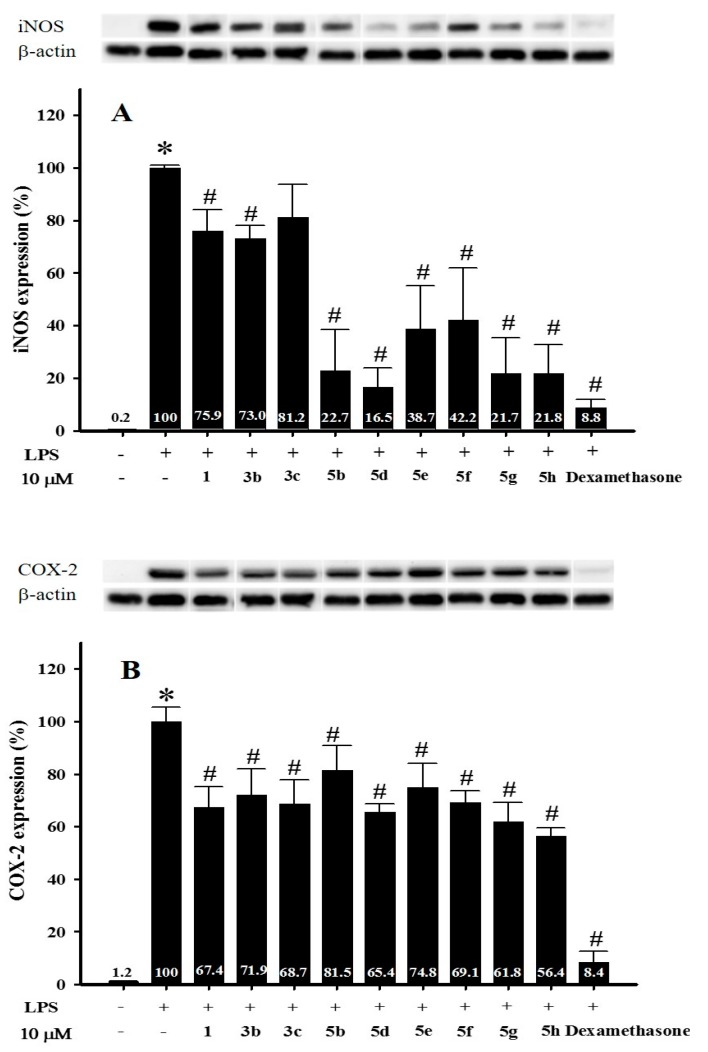
Effect of compounds **1**, **3b**, **3c**, and **5b**–**h** on (**A**) iNOS and (**B**) COX-2 protein expression of LPS-stimulated RAW264.7 macrophage cells by immunoblot analysis. The results are expressed in means ± SEM * *p* < 0.05 and # *p* < 0.05, as compared to the control and LPS-stimulated group, respectively. β-Actin was used as protein equal loading control.

In another experiment, nalidixic acid (**1**) exerted a moderate but significant (*p* < 0.05) downregulation of both iNOS and COX-2 expression to 75.9% ± 9.0% and 67.4% ± 7.6%, respectively, at 10 μM relative to that of the control LPS-stimulated cells (Figure 1). Although the Schiff bases synthesized from isatins with amino acid conjugates of nalidixic acid (**5b** and **5d**–**h**) inhibited both iNOS and COX-2 expression, their anti-iNOS-based anti-inflammatory activity was found to be very pronounced (*cf.* the proximate equal potency of nalidixic acid alone). Therefore, **5b** and **5d**–**h** exhibited powerful reduction in iNOS expression to the level of 16.5% ± 7.4%–42.2% ± 19.6% compared to their inhibitory effect on COX-2 expression (<56.4% ± 3.1% inhibition) at the same concentration (10 μM) (Figure 2). Although l-Leu and l-Phe did strongly reduce the iNOS expression (Figure 1), their conjugates with nalidixic acid (**3b** and **3c**) either maintain or partially reduce the anti-iNOS expression activity of nalidixic acid, respectively (Figure 2) compared to their Schiff bases with isatins **5d**–**h,** which exhibited strong iNOS inhibitory activity. Although neither isatins nor amino acids exhibited significant anti-COX-2-based anti-inflammatory effect (Figure 1), it seems that they maintained the basic COX-2 expression inhibitory activity of nalidixic acid (**1**) in the molecules of **3b**, **3c**, and **5d**–**f** (81.5% ± 9.2%–69.1% ± 4.5% inhibition) (Figure 2). However, a probable synergism in the anti-COX-2-based anti-inflammatory effect may be found in the case of conjugates synthesized from halogenated isatins with l-Phe conjugates of nalidixic acid as observed in **5g** and **5h** (61.8% ± 7.3% and 56.4% ± 3.1% inhibition, respectively). It appears that **5g** and **5h** structures, containing halogenated isatin and l-Phe with nalidixic acid, represent the most active biphasic iNOS and COX-2-based anti-inflammatory agents that may be regarded as a new class of nalidixic acid-based antiinflammtory agents.

## 3. Discussion

In this work, we have tried to analyze the anti-inflammatory activity of isatins and certain l-amino acids with the aim to add an anti-inflammatory potential to the known antibacterial nalidixic acid. This was achieved through the synthesis of new isatin Schiff bases linked to nalidixic acid through an amino acid bridge. The design of this study was based on the following facts.

It is known that isatin (1*H*-indole-2,3-dione) is a plant-derived natural compound, and, in humans, it is a metabolic derivative of adrenaline [30]. It has shown antibacterial, anti-tubercular, and antifungal activities [10,11]. Moreover, it has been reported to inhibit the production of nitric oxide in LPS–interferon-γ–stimulated RAW 264.7 macrophage cells and inhibit the production of prostaglandin E2 (PGE2) and tumor necrosis factor (TNF-α) through its effect on expression of inducible nitric oxide synthase (iNOS) and cyclooxygenase-2 (COX-2), respectively [12]. The same anti-inflammatory-related activities were also observed with isatin analogs e.g., 5-, 6-, or 7-chloroisatin and 5-methylisatin [12]. The synthetic versatility of isatin has also led to its extensive use as a precursor in organic synthesis of interesting molecules with anticancer [31], antiviral [32], anti-HIV [33], and anticonvulsant [34] activities. The incorporation of amino acids was also supposed to improve the possible anti-inflammatory activity of the whole molecule containing nalidixic acid and isatin moieties on the basis of the following facts. BAAs such as Isoleucine and Leucine have been reported to exhibit anti-inflammatory activity, which were thought to be related to the interference with the action and/or synthesis of prostaglandins [13]. A supplementation of BAA mixture (Leucine, Isoleucine, and Valine) effectively reduces the muscle soreness and fatigue sensation, which could be attributed to the attenuation of muscle damage and inflammation [14]. Other amino acids, e.g., Glycine, Histidine and Cysteine, exhibited anti-inflammatory effects in human coronary arterial endothelial cells though inhibition of NF-κB activation and IL-6 production [21]. Some amino acid conjugates with other compounds have been found to create, maintain, or improve the anti-inflammatory effect. Therefore, some N-linked amino acid conjugates with linoleic acid, 2,5-diaryl substituted furans, and substituted 1,3-dioxanes showed potential anti-inflammatory effect [15,16,17]. Moreover, amino acid conjugates (e.g., with the NSAID pirprofen) kept the anti-inflammatory activity with the advantage of a significant elimination of ulcerogenicity as compared to the parent drug [35]. A similar phenomenon was also observed for the NSAID celecoxib bound with amino acids, which showed selective COX-2 inhibitory activity [18].

On the basis of the above findings, the anti-inflammatory activity of the selected l-amino acids, isatin moieties, and nalidixic acid in this study was primarily evidenced. These starting materials exhibited *in vitro* high selective capacity to down-regulate iNOS protein expression in the LPS-stimulated RAW264.7 macrophage cells. Nalidixic acid alone also showed a moderate down-regulation effect but against both iNOS and COX-2 expressions with a balanced potency. As a result, the integrated anti-inflammatory activities of amino acid, isatin, and nalidixic acid moieties were thus disclosed in the newly synthesized Schiff bases (**5b** and **5d**–**h**). The Schiff bases have been shown to inhibit both iNOS and COX-2 expression but with much more powerful anti-iNOS expression effect at the same concentration. The compounds also still retained the antibacterial properties of nalidixic acid [20]. However, it seems that amino acids might have no significant role or merely maintain the anti-iNOS and anti-COX expression activity of nalidixic acid as indicated by comparison of the effect of nalidixic acid alone with that of the tested nalidixic acid–amino acid hydrazide **(3b** and **3c**). Nevertheless, in a molecular docking study, it was found that 5-chloro- and 5-bromoisatin-based hydrazide derivatives possessed good anti-inflammatory activity because of good binding scores into the active site of COX-2 enzyme through hydrogen bond and aromatic interaction with Gln-275 and His-193, respectively [36]. This fact may add another mechanism for the anti-COX-2 activity of the new 5-haloisatin Schiff bases with nalidixic acid-amino acid hydrazide (e.g., **5g** and **5h**). However, these kind of Schiff bases exhibited significant biphasic iNOS and COX-2-based anti-inflammatory activity. Thus, it was concluded that the new synthesized nalidixic acid-based conjugates reported herein could provide suitable templates in a drug discovery program of novel anti-inflammatory antibiotics for promoting the resolution of chronic microbial and/or non-microbial inflammation. However, the effect of these compounds on the cellular mechanisms of iNOS and COX-2 protein expression, e.g., by inhibiting the activation of the nuclear factor (NF)-κB, and on the activity of the two enzymes is worth being investigated in our future studies.

## 4. Materials and Methods

### 4.1. General

The organic solvents and chemicals, including l-configured amino acids, were obtained from Sigma-Aldrich (St. Louis, MO, USA) and Fluka (Buchs, Switzerland). Microwave irradiation was carried out using a microwave oven LG-MS-2044 W/OO (LG Electronics, Beijing, China), with a frequency of 2450 MHz and operating at 420 watts of the total power. IR spectra were recorded on a Perkin Elmer FT-IR spectrometer (Perkin Elmer, Waltham, MA, USA) at College of Pharmacy, King Saud University, and were reported in wave numbers (cm^−1^). Melting points (m.p.) were determined in opened glass capillary tubes with an Electrothermal apparatus IA9100 (Shimadzu, Tokyo, Japan) and are uncorrected. Elemental analysis for C, H and N was measured in Microanalytical Unit, NRC, Cairo, Egypt. Specific optical rotations (${\left[\alpha \right]}_{D}^{25}$) were measured in methanol with an Optronic P8000 polarimeter (A. Krüss, Hamburg, Germany) in a 1 dm length observation tube. NMR spectra were recorded on a Bruker NMR spectrometer (Bruker AXS Inc., Flawil, Switzerland) operating at 500 MHz for ^1^H and 125.76 MHz for ^13^C, at the Research Center, College of Pharmacy, King Saud University. Chemical shifts (δ) are expressed in ppm relative to tetramethylsilane (TMS) as an internal standard and coupling constants (*J*) are expressed in Hz. D_2_O was added to confirm the exchangeable protons. MS were measured on triple quadruple mass spectrometer (Waters, Milford, MA, USA). Thin-layer chromatography (TLC) was performed on Silica gel 60 F_254_ aluminum sheets, (E. Merck, Darmstadt, Germany).

### 4.2. Synthesis of Nalidixic Acid Carboxamides of Amino Acids Esters *(**2a**–**c**)*

To a cold and stirred dry dichloromethane solution (25 mL, −20 °C) of nalidixic acid (1 mmol), ethyl chloroformate (1 mmol) and triethylamine (1 mmol) were successively added. After 10 min, a cold methylene chloride solution (10 mL, −20 °C) of an amino acid methyl ester, namely l-Valine methyl ester, l-Leucine methyl ester, or l-Phenylalanine methyl ester (1 mmol) was added, with continuous stirring for 3 h and at RT overnight. The solution was then washed with water, 1 N HCl, 1 N NaHCO_3_, and finally with water (250 mL). The solution was dried on anhydrous CaCl_2_ and evaporated. The oily residue was solidified by trituration with dry ether, filtered off, dried under vacuum, and recrystallized to afford the esters **2a**–**c**, respectively, as previously described [37].

### 4.3. Synthesis of Hydrazide Derivatives *(**3a**–**c**)*

Hydrazine hydrate 99% (0.8 mL, 16 mmol) was added to a methanolic solution (10 mL) of **2a**–**c** (1 mmol), individually. The reaction mixture was refluxed for 10 h, and then evaporated under vacuum. The residue was triturated with ether, filtered off, and recrystallized to afford the corresponding hydrazides **3a**–**c**. Compound **3a** was previously identified as 1-ethyl-*N*-(1-hydrazinyl-3-methyl-1-oxobutan-2-yl)-7-methyl-4-oxo-1,4-dihydro-1,8-naphthyridine-3-carboxamide [37].

*1-Ethyl-N-(1-hydrazinyl-4-methyl-1-oxopentan-2-yl)-7-methyl-4-oxo-1,4-dihydro-1,8-naphthyridine-3-carboxamide* (**3b**). Yield 63%; m.p. 94–96 °C; ${\left[\alpha \right]}_{D}^{25}$ = −50 (*c* = 0.008, MeOH). IR (KBr): ν_max_ 3381–3055 (NH, NH_2_), 1654 (C=O), 1609, 1539, 1254 (C=O, amide I, II, II) cm^−1^. ^1^H-NMR (500 MHz, (DMSO-*d*_6_): δ 0.9 (6H, t, *J* = 7.5 Hz, 2 × -CH_3_), 1.36 (3H, t, *J* = 7.5 Hz, CH_2_CH_3_),1.63 (2H, q, *J* = 7.0 Hz, -CH-CH_2_-CH), 2.65 (3H, s, -CH_3_ Napth.), 4.44 (2H, m, -NH_2_, D_2_O exchg.), 4.58 (2H, q, *J* = 7.0 Hz, -CH_2_ Napth.), 4.61 (1H, q, *J* = 6.0 Hz, -NHCH), 7.45 (1H, d, *J* = 8.0 Hz, C-6 Napth.), 8.52 (1H, d, *J* = 8.5 Hz, C-5 Napth.), 8.94 (1H, s, C-2 Napth.), 9.35 (1H, s, -NH, D_2_O exchg.), 10.94 (1H, d, *J* = 8.5 Hz, CONH, D_2_O exchg.); ^13^C-NMR (125 MHz, DMSO-*d*_6_): δ 13.2, 15.5, 22.5, 23.4, 24.9, 25.3, 42.8, 46.4, 49.9, 112.3, 120.1, 121.9, 136.3, 148.4, 148.5, 163.6, 163.6, 171.4, 176.2. EI-MS *m*/*z* 359.11 [M^+^]. Anal. calcd. for C_18_H_25_N_5_O_3_ (359.20): C (60.15), H (7.01), and N (19.45). Found: C (60.12), H (7.00), and N (19.44).

*1-Ethyl-N-(1-hydrazinyl-1-oxo-3-phenylpropan-2-yl)-7-methyl-4-oxo-1,4-dihydro-1,8-naphthyridine-3-carboxamide* (**3c**). Yield 67%; m.p. 224–226 °C; ${\left[\alpha \right]}_{D}^{25}$ = −62 (*c* = 0.008 , MeOH); IR (KBr): ν_max_ 3587–3061 (NH, NH_2_), 1734 (C=O), 1607, 1531, 1252 (C=O, amideI, II, II) cm^−1^; ^1^H-NMR (500 MHz, DMSO-*d*_6_) δ 1.36 (3H, t, *J* = 7.5 Hz, -CH_3_), 2.65 (3H, s, -CH_3_ Napth.), 2.90 (1H, q, *J* = 7.0 Hz, -CH_2_CH_3_), 3.04 (1H, q, *J* = 7.0 Hz, -CH_2_CH_3_), 4.26 (2H, s, -NH_2_, D_2_O exchg.), 4.53 (2H, q, *J* = 7.0 Hz, -CH_2_ Ph.), 4.77 (1H, q, *J* = 6.0 Hz, -NHCH), 7.18–7.27 (5H, m, Ar-H), 7.45 (1H, d, *J* = 8.0 Hz, C-6 Napth.), 8.53 (1H, d, *J* = 8.0 Hz, C-5 Napth.), 8.88 (1H, s, C-2 Napth.), 9.32 (1H, s, -NH, D_2_O exchg.), 10.17 (1H, d, *J* = 8.0 Hz, CONH, D_2_O exchg.); ^13^C-NMR (125 MHz, DMSO-*d*_6_) δ 13:2, 15.5, 19.0, 25.3, 46.4, 53.0, 56.5, 112.2, 120.07, 121.9, 126.8, 128.5, 128.6, 129.6, 136.4, 137.9, 148.4, 148.5. EI-MS *m*/*z* 393.01 [M^+^]. Anal. calcd. for C_21_H_23_N_5_O_3_ (393.18): C (64.11), H (5.89), and N (17.80). Found: C (64.10), H (5.87), and N (17.84).

### 4.4. Synthesis of Schiff Bases *(**5a**–**h**)*

A solution of each of isatin **4a**–**c** (1 mmol) and hydrazide **3a**–**c** (1 mmol) in ethanol (15 mL) was prepared. Each of the isatin and hydrazide solutions was mixed, separately, followed by addition of few drops of glacial AcOH. The whole reaction mixture was microwave irradiated at 420 W (~110 °C) for 5–10 min and then cooled. The solid separated was processed as usual [37] to obtain hydrazones **5a**–**h**. Compound **5a** was previously identified as (*E*)-*N*-(1-(2-(2-oxoindolin-3-ylidene)hydrazinyl)-4-methyl-1-oxobutan-2-yl)-1-ethyl-7-methyl-4-oxo-1,4-dihydro-1,8-naphthyridine-3-carboxamide [37].

*(E)-N-(1-(2-(5-Chloro-2-oxoindolin-3-ylidene)hydrazinyl)-4-methyl-1-oxobutan-2-yl)-1-ethyl-7-methyl-4-oxo-1,4-dihydro-1,8-naphthyridine-3-carboxamide* (**5b**). Yield: 90%; m.p. 141–143 °C, (MeOH). ${\left[\alpha \right]}_{D}^{25}$ = −123 (*c* = 0.007, Me OH). IR (KBr): ν_max_ 3432–3364 (3NH), 1731 (C=O), 1648, 1530, 1235 (C=O, amide I, II and III), 1617 cm^−1^; ^1^H-NMR (500 MHz, DMSO-*d*_6_): δ 0.93 (6H, t, *J* = 7.5 Hz, 2 × CH_3_), 1.39 (3H, t, *J* = 7.5 Hz, -CH_2_CH_3_), 2.1 (1H, m, CH_3_-CH-CH_3_), 2.67 (3H, s, -CH_3_ Napth.), 3.68 (1H, s, -NH, D_2_O exchg.), 4.50 (1H, q, *J* = 6.0 Hz, NHCH), 4.58 (2H, q, *J* = 7.0 Hz, CH_2_ Napth.), 6.89–7.49 (3H, m, Ar-H), 7.61 (1H, d, *J* = 6.0 Hz, C-6, Napth.), 8.58 (1H, d, *J* = 8.0 Hz, C-5 Napth.), 8.96 (1H, s, C-2, Napth.), 10.35 (1H, d, *J* = 8.0 Hz, -NH, D_2_O exchg.), 11.14 (1H, s, CONH, D_2_O exchg.); ^13^C-NMR (125 MHz, DMSO-*d*_6_): δ 13.2, 15.5,18.3, 19.6, 25.3, 30.8, 46.6, 52,3, 57.5, 111.9, 114.3, 119.6, 120.1, 122.0, 124.6, 127.6, 136.4, 137.7, 148.6, 148.7, 159.6, 163.7, 164.4, 172.4, 176.5, 183.8. EI-MS *m*/*z* = 508.16 [M^+^]. Anal. calcd. for C_25_H_25_ClN_6_O_4_ (508.13): C (59.00), H (4.95), N (16.51). Found: C (59.31), H (4.60), N (16.62).

*1-Ethyl-N-(1-hydrazinyl-1-oxo-3-phenylpropan-2-yl)-7-methyl-4-oxo-1,4-dihydro-1,8-naphthyridine-3-carboxamide* (**5c**). Yield 88%; m.p. 109–112 °C, (MeOH). ${\left[\alpha \right]}_{D}^{25}$ = −117 (*c* = 0.009, MeOH). IR (KBr): ν_max_ 3428–3353 (3NH), 1722 (C=O), 1643, 1527, 12,392 (C=O, amide I, II and III),16 11 cm^−1^; ^1^H-NMR (500 MHz, DMSO-*d*_6_): δ 1.0 (6H, t, *J* = 7.5 Hz, 2 × CH_3_), 1.39 (3H, t, *J* = 7.5 Hz, -CH_2_CH_3_), 2.2 (1H, m, CH_3_-CH-CH_3_), 2.67 (3H, s, -CH_3_ Napth.), 3.68 (1H, s, -NH, D_2_O exchg.), 4.56 (1H, q, *J* = 7.0 Hz, CH_2_ Napth.), 6.86 (1H, q, *J* = 8.5 Hz, NHCH), 7.40–7.63 (3H, m Ar-H), 7.74 (1H, d, *J* = 7.5 Hz, C-6, Napth.), 8.57 (1H, d, *J* = 8.0 Hz, C-5 Napth.), 8.97 (1H, s, C-2, Napth.), 10.35 (1H, d, *J* = 8.0 Hz, -NH, D_2_O exchg.), 11.14 (1H, s, CONH, D_2_O exchg.); ^13^C-NMR (125 MHz, DMSO-*d*_6_): δ 13.2, 15.5, 18.3, 19.6, 25.3, 30.8, 46.6, 52.3, 57.5, 65.4, 111.9, 114.7, 120.0, 122.0, 127.4, 136.4, 140.5, 148.6,150.0, 159.4, 163.8, 164.4, 172.4, 176.5, 183.7. EI-MS *m*/*z* 552.11 [M^+^]. Anal. calcd. for C_25_H_25_BrN_6_O_4_ (552.18): C (54.26), H (4.55), N (15.19). Found: C (54.29), H (4.60), N (15.20).

*(E)-N-(1-(2-(2-Oxoindolin-3-ylidene)hydrazinyl)-4-methyl-1-oxopentan-2-yl)-1-ethyl-7-methyl-4-oxo-1,4-dihydro-1,8-naphthyridine-3-carboxamide* (**5d**). Yield 83%; m.p. 160–164 °C, (MeOH). ${\left[\alpha \right]}_{D}^{25}$ = −50 (*c* = 0.008, MeOH). IR (KBr): ν_max_ 3413–3197 (3NH), 1701 (C=O), 1607, 1534, 1253 (C=O, amide I, II, II) cm^−1^. ^1^H-NMR (500 MHz, DMSO-*d*_6_): δ 0.94 (6H, t, *J* = 7.5 Hz, 2 × CH_3_), 1.07 (1H, s, -NH, D_2_O exchg.), 1.37 (3H, t, -CH_2_CH_3_), 1.77 (2H, m, CH-CH_2_-CH), 1.91 (1H, s, -CH_3_-CH-CH_3_), 2.63 (3H, s, -CH_3_ Napth.), 4.56 (2H, q, *J* = 7.0 Hz, -CH_2_ Napth.), 5.60 (1H, s, NHCH), 6.87–7.38 (4H, m Ar-H), 7.50 (1H, d, *J* = 7.0 Hz, C-6, Napth.), 8.54 (1H, d, *J* = 8.0 Hz, C-5 Napth.), 8.98 (1H, s, C-2, Napth.), 10.32 (1H, d, *J* = 7.5 Hz, -NH, D_2_O exchg.), 11.27 (1H, s, CONH, D_2_O exchg.); ^13^C-NMR (125 MHz, DMSO-*d*_6_): δ 13.2, 15.5, 21.6, 21.8, 24.5, 25.3, 42.7, 46.6,49.9, 53.0, 113.2, 118.3, 120.1, 121.9, 123.5, 128,7, 129.6, 129.7, 131.7, 136.4, 148.5, 148.7, 162.8, 163.8, 172.9, 167.4. EI-MS *m*/*z* 488.22 [M^+^]. Anal. calcd. for C_26_H_28_N_6_O_4_ (488.13): C (63.92), H (5.78), N (17.20). Found: C (63.01), H (5.60), N (17.62).

*(E)-N-(1-(2-(5-Chloro-2-oxoindolin-3-ylidene)hydrazinyl)-4-methyl-1-oxopentan-2-yl)-1-ethyl-7-methyl-4-oxo-1,4-dihydro-1,8-naphthyridine-3-carboxamide* (**5e**). Yield 79%; m.p. 164–167 °C, (MeOH). ${\left[\alpha \right]}_{D}^{25}$ = −48 (*c* = 0.01, MeOH). IR (KBr): ν_max_ 3412–3185 (3NH), 1700 (C=O), 1654, 1537, 1253 (C=O, amide I, II, II) cm^−1^. ^1^H-NMR (500 MHz, DMSO-*d*_6_): δ 0.93 (6H, t, *J* = 7.5 Hz, 2 × CH_3_), 1.04 (1H, s, -NH, D_2_O exchg.), 1.39 (3H, t, *J* = 7.5 Hz, -CH_2_CH_3_), 1.70 (2H, m, CH-CH_2_-CH), 1.90 (1H, s, -CH_3_-CH-CH_3_), 2.70 (3H, s, -CH_3_ Napth.), 4.56 (2H, q, *J* = 7.0 Hz, -CH_2_ Napth.), 4.65 (1H, q, *J* = 6.0 Hz, NHCH), 6.91–7.40 (3H, m Ar-H), 7.50 (1H, d, *J* = 7.5 Hz, C-6, Napth.), 8.57 (1H, d, *J* = 7.5 Hz, C-5 Napth.), 8.98 (1H, s, C-2, Napth.), 10.39 (1H, s, -NH, D_2_O exchg.), 11.39 (1H, s, CONH, D_2_O exchg.); ^13^C-NMR (125 MHz, DMSO-*d*_6_)): δ 13.2, 15.4, 21.5, 21.8, 23.6, 25.3, 42.7, 46.6, 50.0, 53.0, 113.2, 118.3, 120.1, 121.9, 123.5, 128,7, 129.6, 129.7, 131.7, 136.4, 148.5, 148.6, 162.8, 163.8, 172.5, 167.4. EI-MS *m*/*z* 522.18 [M^+^]. Anal. calcd. for C_26_H_27_ClN_6_O_4_ (522.31): C (59.71), H (5.20), N (16.07). Found: C (59.40), H (5.60), N (16.02).

*(E)-1-Ethyl-7-methyl-4-oxo-N-(1-oxo-1-(2-(2-oxoindolin-3-ylidene)hydrazinyl)-3-phenylpropan-2-yl)-1,4-dihydro-1,8-naphthyridine-3-carboxamide* (**5f**). Yeild 88%%, m.p. 182–185 °C., ${\left[\alpha \right]}_{D}^{25}$ = −208 (*c* = 0.024, MeOH). IR (KBr): ν_max_ 3420–3219 (3NH), 1720 (C=O), 1609, 1540, 1255 (C=O, amide I, II, II) cm^−1^. ^1^H-NMR (500 MHz, DMSO-*d*_6_): δ 1.36 (3H, t, *J* = 7.0 Hz, -CH_3_), 1.90 (1H, s, -NH, D_2_O exchg.), 2.66 (3H, s, -CH_3_ Napth.), 3.25 (2H, s, CH_2_CH_3_), 4.52 (2H, q, *J* = 7.0 Hz, -CH_2_Ph.), 5.80 (1H, s, NHCH), 6.90–7.45 (9H, m Ar-H), 7.5 (1H, d, *J* = 7.0 Hz, C-6, Napth.), 8.57 (1H, d, *J* = 6.0 Hz, C-5 NApth.), 8.94 (1H, s, C-2, Napth.), 10.40 (1H, s, -NH, D_2_O exchg.), 10.83 (1H, s, CONH, D_2_O exchg.); ^13^C-NMR (125 MHz, DMSO-*d*_6_) δ 13:2, 15.5, 21.5, 25.3, 38.1, 46.5, 111.1, 112.7, 115.7, 118.3, 120.1, 122.0, 123.2, 125.1, 126.7, 127.1, 128.7, 129.7, 129.8, 136.4, 137.4, 138.8, 144.4, 148.6, 151.2, 159.8, 163.7, 165.1, 172.5, 176.3. EI-MS *m*/*z* 522.14 [M^+^]. Anal. calcd. for C_29_H_26_N_6_O_4_ (522.20): C (66.66), H (5.02), N (16.08). Found: C (66.63), H (5.10), N (16.11).

*(E)-N-(1-(2-(5-Chloro-2-oxoindolin-3-ylidene)hydrazinyl)-1-oxo-3-phenylpropan-2-yl)-1-ethyl-7-methyl-4-oxo-1,4-dihydro-1,8-naphthyridine-3-carboxamide* (**5g**). Yield 81%; m.p. 197–200 °C, (MeOH). ${\left[\alpha \right]}_{D}^{25}$ = −250 (*c* = 0.016, MeOH). IR (KBr): ν_max_ 3420–3180 (3NH), 1718 (C=O), 1654, 1522, 1253 (C=O, amide I, II, II) cm^−1^. ^1^H-NMR (500 MHz, DMSO-*d*_6_)): δ 1.36 (3H, t, *J* = 7.5 Hz, -CH_3_), 1.91 (1H, s, -NHCH, D_2_O exchg.), 2.66 (3H, s, -CH_3_ Napth.), 3.0 (1H, NHCH), 3.25 (2H, s, CH_2_CH_3_), 4.55 (2H, q, *J* = 7.0 Hz, -CH_2_Ph.), 6.90–7.36 (8H, m Ar-H), 7.46 (1H, d, *J* = 8.0 Hz, C-6, Napth.), 8.57 (1H, d, *J* = 3.5 Hz, C-5 Napth.), 8.93 (1H, s, C-2, Napth.), 10.47 (1H, s, -NH, D_2_O exchg.), 10.0 (1H, s, CONH, D_2_O exchg.); ^13^C-NMR (125 MHz, DMSO-*d*_6_): δ 13.2, 15.4, 21.5, 25.3, 38.0, 46.5, 57.8, 111.5, 113.1, 116.7, 120.2, 120.4, 121.9, 122.0, 124.5, 126.2, 127.1, 127.7, 129.7, 129.8, 136.4, 137.4, 137.9, 143.1, 148.6, 163.7, 164.9, 172.5, 176.3. EI-MS *m*/*z* 556.16 [M^+^]. Anal. calcd. for C_29_H_25_ClN_6_O_4_ (556.19): C (62.53), H (4.52), N (15.09). Found: C (62.40), H (4.50), N (15.11).

*(E)-N-(1-(2-(5-Bromo-2-oxoindolin-3-ylidene)hydrazinyl)-1-oxo-3-phenylpropan-2-yl)-1-ethyl-7-methyl-4-oxo-1,4-dihydro-1,8-naphthyridine-3-carboxamide* (**5h**). Yield 85%; m.p. 188–190 °C, (MeOH). ${\left[\alpha \right]}_{D}^{25}$ = −125 (*c* = 0.008, Me OH). IR (KBr): ν_max_ 3414–3180 (3NH), 1718 (C=O), 1607, 1533, 1252 (C=O, amide I, II, II) cm^−1^. ^1^H-NMR (500 MHz, DMSO-*d*_6_): δ 1.36 (3H, t, *J* = 7.5 Hz, -CH_3_), 1.90 (1H, s, -NHCH, D_2_O exchg.), 2.66 (3H, s, -CH_3_ Napth.), 3.0 (1H, NHCH), 3.25 (2H, s, CH_2_CH_3_), 4.54 (2H, q, *J* = 7.0 Hz, -CH_2_Ph.), 6.80–7.50 (8H, m Ar-H), 7.63 (1H, d, *J* = 7.0 Hz, C-6, Napth.), 8.56 (1H, d, *J* = 8.0 Hz, C-5 Napth.), 8.93 (1H, s, C-2, Napth.), 10.46 (1H, s, -NH, D_2_O exchg.), 10.90 (1H, s, CONH, D_2_O exchg.); ^13^C-NMR (125 MHz, DMSO-*d*_6_)): δ 13.2, 15.5, 21.5, 25.3, 38.0, 46.5, 56.5, 112.7, 113.6, 114.8, 117.2, 120.1, 122.0, 122.3, 123.7, 127.2, 127.3, 128.8, 129.7, 129.8, 136.4, 140.5, 143.4, 148.6, 150.0, 159.4, 163.7, 164.7, 172.5, 176.3. EI-MS *m*/*z* 600.11 [M^+^]. Anal. calcd. for C_29_H_25_BrN_6_O_4_ (600.16): C (57.91), H (4.19), N (13.97). Found: C (57.52), H (4.50), N (13.70).

### 4.5. Cell Viability Assay

The RAW264.7 macrophage cells obtained from the American Type Culture Collection (ATCC, #TIB-71) were cultured on 96-well plates. The testing compound at 10 mM was incorporated into the cell culture in DMEM medium in humidified incubator within a 5% CO_2_ atmosphere at 37 °C for 24 h. AlamarBlue^®^ (Invitrogen, Carlsbad, CA, USA) was added according to the manufacturer instruction, the absorbance (A) was measured at 570 nm, and the cell viability was then analyzed [38].

### 4.6. In Vitro Anti-inflammatory Assay

The method described by Ho *et al.* [39] was modified by our group [22] and employed to measure the *in vitro* anti-inflammatory activity of amino acids, isatins (**4a**–**c** ), and the synthesized compounds (**3b**, **3c**, **5b** and **5d**–**h**) in comparison with dexamethasone, a positive control. Briefly, cultured RAW264.7 cells were incubated with LPS (0.01 mg/mL) in the presence (10 μM) or absence of the compounds. The intracellular protein containing-supernatant fraction was then used for measuring COX-2 and iNOS protein expression using sodium dodecyl sulfate polyacrylamide gel electrophoresis (SDS-PAGE) and immunoblot analysis. β-actin was used as an internal loading control.

## 5. Conclusions

A series of nalidixic acid, amino acid, and isatin-based Schiff bases have been synthesized and evaluated for their anti-inflammatory activity. The compounds showed an anti-inflammatory effect via inhibition of the proinflammatory iNOS and COX-2 proteins expression with variable potencies. This is the first report to point out that the newly synthesized linear macromolecules of nalidixic acid linked to isatin via certain l-amino acid bridges can be regarded as a novel class of anti-inflammatory antibacterial agents.

## Abbreviations

The following abbreviations are used in this manuscript:

BAA	Branched amino acids
COX-2	Cyclooxygenase-2
iNOS	Inducible nitric oxide Synthase
LPS	*E. coli* lipopolysaccharide
NSAIDs	nonsteroidal antiinflammatory drugs
NMR	Nuclear magnetic resonance
MW	Microwave
